# Impact of multiprofessional collaboration in primary care practices on continuity of care: a scoping review

**DOI:** 10.1186/s12875-026-03507-y

**Published:** 2026-08-03

**Authors:** Sebastian Kegreiss, Patricia Lampart, Stefan Essig, Rebecca Tomaschek, David Schiltknecht, Stefan Neuner-Jehle

**Affiliations:** 1 Center for Primary and Community Care, University of Lucerne, Lucerne, Switzerland; 2 Institute of Primary Care, University of Zurich and University Hospital Zurich, Zurich, Switzerland

**Keywords:** Continuity of care, Care continuity, Primary health care, Primary care, Multiprofessional care

## Abstract

**Background:**

Multiprofessional care in Primary Care practices is increasingly common. However, the presence of multiple professional roles in the practice team may affect continuity of care (CoC), whether through newly established multiprofessional teams or changes within existing team structures. CoC has been shown to decline in recent years contrasting to its core value for primary care. This scoping review aims to determine the effect of multiprofessional patient care within the primary care practice team on CoC, and to evaluate the extent and type of evidence.

**Methods:**

A literature search was conducted using the databases PubMed, CINAHL, Embase, and the Cochrane Library. Peer-reviewed, original articles with either quantitative, qualitative or mixed methods design, with no limit on publication date and written in any language were considered. The search strings combined the three concepts: primary care, CoC and multiprofessional co-location. Studies were included if they examined any effect of multiprofessional care in a practice-based primary care setting on CoC, using a stepwise and hierarchical screening procedure of titles, abstracts and full-texts. Studies were analysed according to Haggerty et al.’s framework of informational, relational, and management domains of CoC.

**Results:**

The initial search resulted in 6525 unique studies. The final dataset included 21 studies utilising data from 2010 onward. The studies suggest that CoC may suffer if care is provided by multiple professionals, foremost the relational domain of CoC, whereas informational and management CoC were generally maintained or even improved. For the relational CoC, expectations and concerns in regard with introducing new professional roles in the primary care practice varied between providers and patients, with more pronounced concerns among patients. Small, stable teams and practice sizes with strong communication were consistently mentioned as key facilitators of CoC.

**Conclusion:**

The relationship between CoC and multiprofessional care is complex and context dependent. When new professional roles are introduced in primary care practice teams, multiprofessional care may offer the opportunity to maintain or even improve CoC. Importantly, expectations and concerns of providers and patients must be considered and addressed, especially for relational CoC which is a major concern for patients in the context of multiprofessional care.

**Supplementary Information:**

The online version contains supplementary material available at https://doi.org/10.1186/s12875-026-03507-y.

## Introduction

Primary care systems in many other countries are facing an increasing demand due to rising patient loads and the growing complexity of patient care. At the same time, many studies document a perceived shortage of primary care physicians (PCPs) and PCPs working fewer hours per week on average, with increasingly less time spent on patient care and increasingly more time on administrative tasks [1, 2]. Furthermore, primary care practices are moving away from single-handed practices run by full-time PCPs towards practices with larger teams and more professions [1, 3].

In the context of the primary care practice, continuity of care (CoC) is an essential concept and goal. CoC is a complex construct and closely related to and entangled with coordination of care, integration of care and patient-centered care. Understanding of the concept has changed over time. While CoC was initially understood as having a personal care provider a patient seeing the same PCP, later multidimensional models were introduced to define CoC [4]. One of the most frequently cited models was developed by Haggerty et al. [5] who describe three domains of CoC: *Informational CoC* refers to the use of disease- or person-focused information linking care across providers and healthcare events. It comes in written form and focuses on a person’s medical condition. Furthermore, knowledge of a patient’s preferences, values, and personal context is equally important for ensuring this type of CoC across care encounters and is often retained in the memory of individual providers. *Relational CoC* describes a therapeutic relationship between a patient and one or more caregiver. It not only bridges past to current care but also provides a link to future care which plays a key role in primary care. *Management CoC* refers to a consistent and coherent management of a person’s health condition that remains responsive to evolving needs. This may be crucial in chronic and complex conditions requiring multiple care providers. It is achieved when services are delivered in a complementary, coordinated and timely manner. Due to its simplicity and widespread use, we will rely on this framework by Haggerty et al. in this study.

CoC in all of its domains is highly valued by patients and is considered a core value in primary care [5]. Transferring primary care tasks to other professionals may relive the strain on primary care systems, with non-physician professionals not simply acting as substitutes for PCPs but taking on new and complementary roles. However, it has been noted that CoC has been declining in recent years [6]. Given the ongoing expansion of multiprofessional primary care teams, it is important to understand how changes in practice organisation may affect CoC. A previous scoping review by the authors found that PCPs working part-time may not offer the same CoC as full-time clinicians [7]. Involving several clinicians in the care of one patient may further reduce the CoC experienced by patients [8]. Decreasing CoC is of concern, since higher CoC has been linked to hard outcomes such as reduced acute hospital admissions, need for out-of-hours services and mortality in a dose-dependent way [9–11].

The objective of this scoping review is a better understanding of how multiprofessional patient care within primary-care practice teams affects CoC, whether through newly established multiprofessional teams or changes within existing team structures.

## Methods

To answer our research question, we conducted a scoping review following the guidelines of the PRISMA-ScR checklist (cf. supplementary material). A scoping review was the appropriate study design to support our aim as the heterogenous concept of CoC does not allow us to pool quantitative results. A protocol for the review was pre-registered with the Open Science Framework on 27 February 2025 [12].

### Literature search

A systematic search was conducted using the following four databases: PubMed, EMBASE, Cinahl, and the Cochrane Library. The search string used a combination of controlled vocabulary, and free-text terms with Boolean operators. The search strings combined three concepts: primary care, continuity of care and multiprofessional co-location (cf. supplementary material). Finally, a hand search was performed based on the reference lists of selected articles.

### Inclusion criteria

Peer-reviewed, original articles with either quantitative, qualitative, or mixed methods designs, with no limit on publication date, written in any language were considered for inclusion. Studies were included if they examined any effect of multiprofessional care in a practice-based primary care setting on CoC. Studies were not required to explicitly use the exact term “continuity”, as long as they reported an outcome aligning with this framework. A PCP was understood to be a physician providing primary care, which is first-contact, accessible, continuous, comprehensive, and coordinated person-focused care as defined by the WHO [13].

Articles were excluded if (1) the multiple professions were not part of the same primary-care practice team (2), CoC was not examined as an outcome, or reported as the outcome of a different intervention than that of multiprofessional patient care within the primary-care practice team (3), practice-based primary care was not the setting (4), the full text was unobtainable (5), the article was not a peer-reviewed original article, i.e. essays, comments, opinions, editorials, poster/conference abstracts, or (6) there was insufficient information reported in the article to answer our research question.

### Selection

The literature search was initially performed on 12.12.2024 and updated on 16.11.2025. Selection was performed in two steps. First, titles and abstracts were screened by two researchers (SK and PL). Discrepancies in the selection choices were assessed by a third researcher (RT or SE). Articles were screened manually without software tools. Second, all full texts of the included abstracts were screened by the first author and independently assessed by a second team member, with the remaining researchers dividing them among themselves to ensure each study was reviewed by two researchers (PL, RT, SE, SNJ). Disagreements were resolved through discussion within the research team, with inclusion or exclusion decisions made by consensus. All researchers involved have previous experience undertaking evidence reviews.

### Data charting

Data collected in the selected articles were extracted and systematically organized in a table (cf. supplementary material). That table contained the following information: title, first author, year of publication, location and setting, study design, population, and outcomes. This process was performed by the first author, followed by a reevaluation with the other researchers.

## Results

### Study selection

**Fig. 1:**
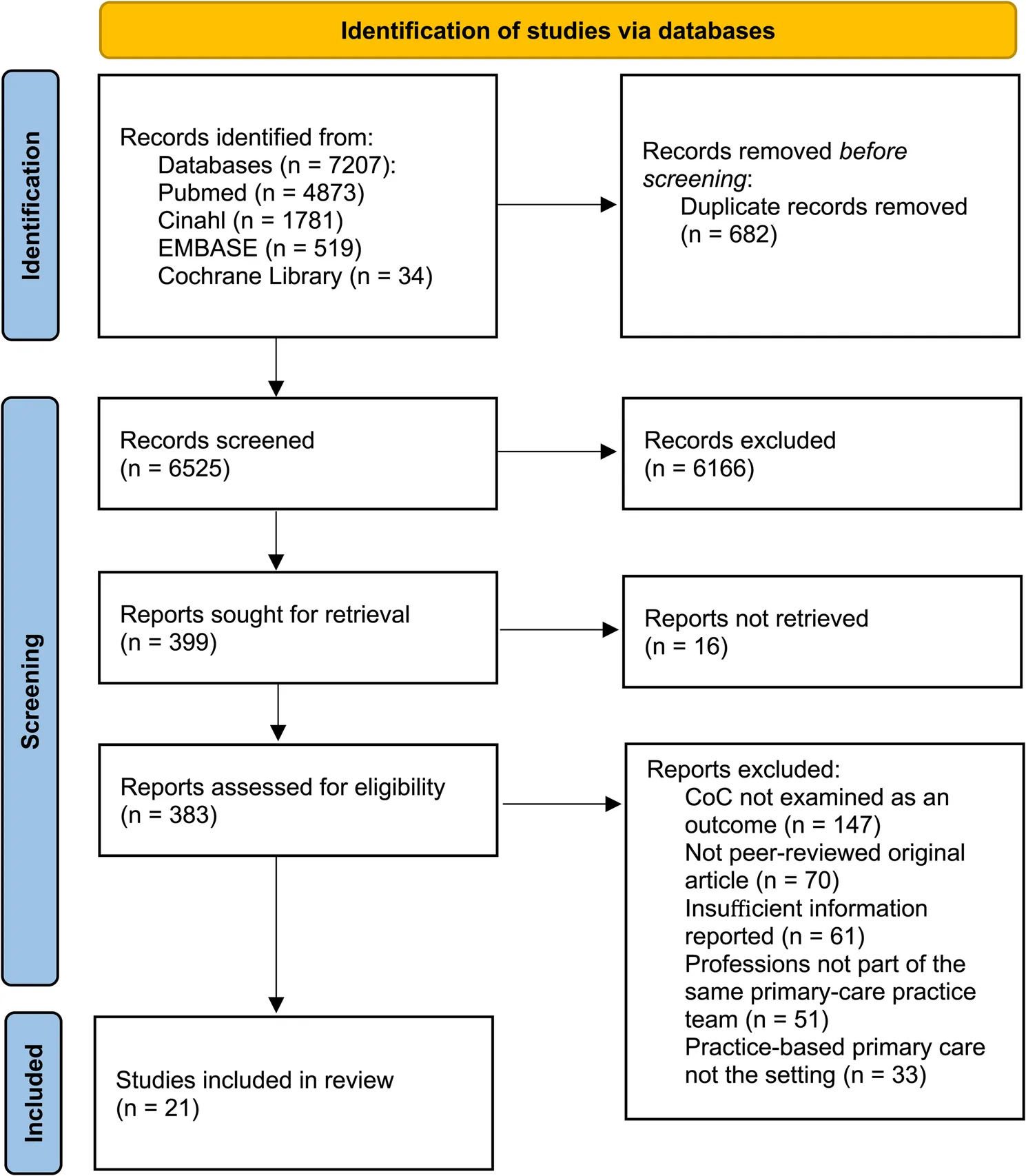
PRISMA Flowchart

As shown in Fig. 1, the search identified 6525 potentially eligible publications after removing duplicates. After screening titles and abstracts, 399 full texts were assessed and 21 studies were included for further analysis.

### Description of included studies

The included studies were published between 2010 and 2025. Five were conducted in the United States of America [14–18], seven in Canada [19–24], one in Australia [25], two in the Netherlands [26, 27], two in the United Kingdom [3, 28], one in Sweden [29], one in Norway [30], one in Singapore [31] and one in Switzerland [32]. One study was conducted in 31 European countries, Canada and New Zealand [33]. All studies discussed interprofessional team-based care involving PCPs and different forms of nursing professionals (i.e. advanced practice nurses (APNs), registered nurses (RNs), licensed practical nurse, advanced clinical/care practitioners, nurse supervisors). Other professions discussed included practice assistants and administrative/supportive staff [3, 15–17, 21, 25, 27, 28, 31], pharmacists [3, 21, 28, 33], social workers [21, 23, 33], practice managers [3, 18, 25], nutritionists or dieticians [19, 20], an unspecified mix of “other allied health professionals” [19, 28], paramedics [3, 28], mental health professionals [21, 23], case managers [16, 18], midwives [33], physiotherapists [33], dentists [33], social prescribers [3] and peer helpers [23]. Study populations directly included patients [16, 20–24, 26, 30–33]. PCPs [3, 15–19, 24, 25, 27, 31, 33], nurses [3, 14, 16–18, 25, 27, 29, 31], practice assistants and administrative/supportive staff [3, 15–17, 25, 27, 31], practice managers [3, 18, 25, 31], clinical pharmacists, paramedics and social prescribers [3].

### Narrative synthesis

Analysis of the included studies revealed substantial heterogeneity in both study design and the measurement of CoC. To facilitate comparison, quantitative studies were grouped according to the principal continuity of care measurement approach employed (Usual Provider Index derived measures, Primary Care Assessment Tool derived measures and large-scale patient surveys), as these instruments assess different quantitative aspects of CoC. Qualitative and mixed-methods studies were grouped according to the perspectives explored (patients versus healthcare professionals). Within each section, findings were synthesised according to Haggerty et al.‘s informational, relational, and management domains of continuity of care [6].

### Usual provider index (relational CoC)

Four studies used indexes similar to or derived from the Usual Provider Index (UPC) to measure relational CoC [16, 17, 22, 26]. The UPC is calculated using the number of a patients visits with the usual PCP divided by the total number of visits to a practice [34], with a higher UPC indicating higher relational CoC.

In a randomized controlled trial among COPD patients in the Netherlands, the UPC Index was significantly higher for patients receiving usual PCP-only care (0.74, 95% CI: 0.61–0.86) compared with those in self-management (0.36, 95% CI: 0.25–0.47, *p* = 0.00) or receiving regular monitoring by a practice nurse (0.50, 95% CI: 0.39–0.61, *p* = 0.02). However, no significant differences in team CoC were observed between the three care models over a two-year period (range of estimates: − 0.21 to 0.12, all *p* > 0.87). Team CoC was understood as CoC provided by the entire primary healthcare team. It was calculated by having patients answering six questionnaire-items relating to management and informational CoC, such as providers team-communication, connectedness, consistency in medical advice, coordination, and shared knowledge of the patient’s history as well as previous visits [26].

A Canadian study by Tourigny et al. reported a significant decrease (*p* < 0.01) in visits to a regular physician compared to the total number of visits to any physician of the clinic [22]. They evaluated how implementation of Family Medical Groups (FMGs), which aimed to promote interprofessional and interorganizational collaborative practices, affected patient’s experiences defined by core dimensions of primary care. FMGs consisted of 8–10 PCPs collaborating with 1 to 2 nurses who offer primary care services including patient follow-up, health promotion and preventative care to a set of registered patients. The study’s intervention included specific attributes to maintain CoC including linking patients to PCPs and professional teams of their FMGs, improvements in electronic health records and care pathways for interdisciplinary follow up.

By contrast, a U.S. military pilot study implementing a team-based, patient-centered primary care model showed improvements in relational CoC. The pilot group showed a significantly higher probability of providers ability to see own patients (72.7% vs. 51.4%, *p* = 0.006) and a non-significant increase in average monthly probability of patients’ ability to see their own provider (73.7% vs. 64.0%, *p* = 0.12). The pilot introduced a “teamlet” of a PCP, a registered nurse (RN), and a RN case manager, each equally responsible for the empaneled population. It also introduced the “My RN” concept, assigning each patient a specific nurse to strengthen CoC [16].

In a large US primary care practice, CoC was operationalized as the proportion of appointments seen by a provider within the patient’s care team (ASCOT), with a higher ASCOT indicating higher CoC. An optimization model demonstrated that balanced teams of 3–4 physicians and NP/PAs achieved consistent ASOCT levels of 62%. Also, having fewer and larger teams increased ASCOT (3% increase when the number of care teams is reduced by 1), as larger teams enabled providers to cover for each other. However, the study noted that this rise in ASCOT may not indicate true CoC. Larger teams increase total patient load which adds more patients to study and manage. In turn, this may undermine team CoC [17].

### Primary care assessment tool (management, informational and relational CoC mixed)

Four studies employed the Primary Care Assessment Tool (PCAT) [21, 24] or an instrument derived from it [22, 23]. The PCAT is a validated, patient-reported measure of primary care performance that includes a CoC (“ongoing care”) subscale, assessing patients’ perceptions of how well their usual primary care providers know them (relational CoC) [35]. Modified versions include informational [22] or management domains [23].

Two studies reported a decrease in relational CoC scores, if primary care teams were larger and several professions were introduced: Kristjansson et al. found that the score decreased with an increase in the total number of providers (effect size = − 0.33%). It also decreased with the number of nurses (effect size = − 0.64%) [24]. Moe et al. found a significant decrease (*P* < 0.001) in relational CoC using PCAT following the introduction of collaborative teams and integrating non-physician health care professionals into community family practices, with the 2 clinics scoring highest in the survey being non-participating solo practices and the lowest-scoring clinic being the one with the largest number of physicians [21]. In contrast, the study by Tourigny et al., which had introduced specific measures to improve CoC reported increased PCAT informational and relational CoC scores (*p* < 0.1) [22]. A Canadian study by Duhoux et al. evaluating patient experiences with Integrated Primary Care Teams (IPCTs, defined by 4 characteristics: intensive interdisciplinary practice; advanced nursing practice with an expanded role; group practice and increased proximity and availability) found variable PCAT CoC scores (relational and management domain) with very little change over time in all IPCTs and suggested that group practice is complex and its reflection in care experience not obvious [23].

### Quantitative large scale surveys with patients (relational CoC)

Three included studies used data from large scale surveys measuring relational CoC. The QUALICO-PC study, a multinational survey of general practitioners and patients in 34 countries (7183 PCPs and 61931 patients), assessed CoC by asking how long patients were known by the PCP [33]. A Canadian Health Services Patient Survey (*n* = 10,868 patients) calculated CoC as the percentage of patients who reported seeing their preferred PCP most of the time [28]. The third study used five English practice-level datasets (*n* = 6,024 general practices) and measured CoC using a Likert-type scale rating how frequently patients received care from the same PCP or nurse [20].

In all three studies, relational CoC was consistently poorer in larger, team-based primary care settings compared with smaller, GP-led practices. In the English study, medium-sized multidisciplinary practices demonstrated a lower CoC score compared to small GP-dependent practices (32.6% vs. 41.2%, *p* < 0.001), while large multidisciplinary practices exhibited a further reduction (27.0% vs. 41.2%, *p* < 0.001) [28]. Patients of singlehanded general practices reported higher perceived CoC than those whose GPs were co-located with other professionals or within multiprofessional teams (*p* < 0.01), with particularly pronounced declines when GPs were co-located with nurses (*p* < 0.001) [33]. In the Canadian survey, team-based primary health care was associated with slightly higher raw CoC scores (mean 3.62 vs. 3.59), but regression analyses indicated a significantly negative association (OR 0.70, *p* < 0.01) [20].

### Qualitative and mixed methods studies with patients

Five studies used interviews [27, 31, 32] or mixed methods (interviews [30], patient-survey [3, 30] and/or focus groups [3]) to evaluate patient perceptions of CoC when general practice shifted from single-profession PCP care to interprofessional models.

#### Management CoC

In a mixed methods study by Abelsen et al. patients perceived management CoC as strengthened, as they found follow-ups being more deliberately planned, care feeling more individualized and better coordinated, consultations to be longer and patients described greater understanding and mastery of their conditions [30].

#### Informational CoC

In the same study by Abelsen informational CoC was reported as improved by patients as PCPs and nurses in the practice team were perceived to share patient information effectively [30]. In contrast patients in the study by Kumar were dissatisfied with receiving conflicting information from different healthcare providers [31].

#### Relational CoC

Patients felt CoC in their relationship with their PCP and reported that nurses strengthened the patients’ link between them and the PCP in the Abelsen study. Relational CoC was also perceived to be strengthened by patients in the study by Kumar et al. which favored having one primary care team eliminating the need to constantly repeat their health history to new personal [31].

Barriers to relational CoC were mentioned in the second mixed-methods study [3]. They found follow-up appointments with a single practitioner to be more complicated to obtain in multiprofessional practices. In the focus groups, patients felt that higher skill mix did not always accommodate relational CoC and reference was made to the tension between enabling CoC and prompt access to appointments with practitioners who might not be their usual practitioner. Patients expressed concern about disrupting long-standing relationships and about the effort required to understand, access, and build relationships with new practitioners. Importantly, several studies suggested that patients distinguished between seeing additional practitioners and seeing new types of practitioners. While patients were often willing to consult nurses or advanced practice nurses, concerns were frequently related to understanding these emerging roles and how they complemented rather than replaced the patient’s relationship with their usual PCP [3, 32]. Patients were generally happy to be seen by newer types of practitioners, but they highlighted the value of CoC and the importance of feeling that new types of practitioners were adequately supported and supervised [3]. This sentiment was also echoed in two interview based studies: patients emphasized the importance of retaining a strong relationship with a PCP: Having “their own” PCP was viewed as central to their concept of CoC [27] and some patients were reluctant to allow APNs to play a leading role in their care and preferred to receive additional confirmation from their PCP, especially with symptoms that were perceived as medically severe [32]. On the other hand, patients may believe that APNs could relieve PCPs’ workload and thereby ensuring CoC [32].

In another study, patients acknowledged how measures such as electronic health records help to ensure informational CoC. However it was not seen as relevant as having a strong personal contact / a relationship with a PCP [27].

### Qualitative studies with health care professionals

Nine studies used interviews [3, 14, 15, 18, 19, 25, 31] or focus groups [27, 29, 31] with health care professionals (HCPs) to obtain qualitative data on all domains of CoC. Three studies reported that introducing multiprofessional team-based care into primary health care practice directly improved CoC, without specifying a particular domain. This effect was even more pronounced with increasing numbers of patients and complexity of care [14, 29, 31].

#### Management CoC

HCPs in several studies talked about management CoC [14, 15, 18, 19, 29, 31]. One study reported that co-locating multiple professions within the same practice could reduce referral to third parties and enable them to get all care from the same practice [18]. Several studies mentioned that non-PCP team members were involved in coordinating patient care [14, 15, 18, 19, 29], for example RNs help to ensure that no important care management tasks are left undone or omitted [14]. Coordination was seen as improved when team members were on-site and readily available [19]. HCPs in one study mentioned that working in a team made them feel more accountable and committed to documenting and managing as other team members needed to follow. Task delegation and redistribution was perceived to ensure seamless care coordination [31]. In a Swedish study, nurse care managers saw themselves as a “spider in the web”, a control center between various contacts [29].

In two studies, nurses were involved in patient follow up, either by telephone [14, 29] or in person [14]. Participants perceived that this enhanced management CoC [14, 29] and that this helped keeping an overview which led to better CoC between providers [29].

#### Informational CoC

CoC was seen as improved if teams were small and stable, and if teams had good team-communication [27], whereas lack of stability and larger team sizes were seen as detrimental [31]. Two studies reported co-location as a facilitator for team-communication [18, 19] as it helped to forge relationships between providers and allowed for face-to-face communication [19]. Additional team members could potentially provide additional perspective and information about the patient [15, 25].

PCPs cautioned that delegating tasks may be a barrier to CoC. Delegation may cause PCPs to lose direct involvement in patient care [27] and could lead to miscommunication and conflicting medical advice [19].

#### Relational CoC

In contrast, HCPs in several studies suggested that task delegation in team-based care could improve relational CoC by making patients’ own PCPs more available. For example, other HCPs could take over care or administrative work and “get basic care duties off the PCPs plate”. This would allow PCPs to spend more time with patient centered care [14, 15, 27].

Two studies found that HCPs viewed relational CoC as being provided by the whole team rather than just the patient’s own GP [25, 27]. It was suggested that patients being too dependent on only one provider may in fact be harmful, as patients may refuse to see other providers if their PCP is unavailable [25]. This could even lead to delay in crucial medical treatment. Two studies expressed comparable views, suggesting other team members may enhance CoC by acting as “relational extensions” and providing an extra link to the PCP [15, 29].

## Discussion

### Main findings

Findings of the studies included reveal many effects of multiprofessional patient care within primary care practice teams on CoC. Quantitative studies often reported that introducing skill-mix may pose risks to CoC, particularly those studies relying on conventional measures of CoC such as the UPC, PCAT, and those employing large-scale patient surveys. Qualitative studies draw a more nuanced picture: although barriers to CoC were mentioned both health care professionals and patients saw the impact of skill-mix more positively than quantitative results suggested.

### Differences by domains of CoC

Table 1 summarizes the main findings regarding the effects of multiprofessional patient care within primary-care practice teams on relational, informational, and management continuity.

**Table 1:** Summary of main findings

CoC Domain	Overall Effect	Main findings
Relational continuity	Mixed / Often negative	Quantitative: Reduced [22, 26] or improved UPC Index [16]. Large scale surveys all report poorer relational CoC [20, 28, 33]. PCAT scores: Poorer in two studies [21, 24], unchanged [23], or improved [22]. Qualititative: Delegation mentioned as facilitator of relational CoC through improved availability of usual PCP [14, 15, 27, 32]. HCPs view relational CoC as provided by entire team [15, 25, 27, 29]. Patients prefer strong relationship with usual PCP [27, 32] or express concerns regarding loss of this relationship [3].
Informational continuity	Neutral to positive	Quantitative: No differences in sub scores relating to informational CoC of team CoC questionnaire [26] and positive PCAT scores [22]. Qualitative: Nurses reported as facilitators of informational CoC [14, 30]. Miscommunication between multiple providers [19, 31] and delegation leading to loss of overview [27] mentioned as barriers to informational CoC.
Management continuity	Neutral to positive	Quantitative: No differences in sub scores relating to management CoC of team CoC questionnaire [26] and neutral PCAT scores [36] Qualitative: HCPs found new professionals in coordinative tasks and patient follow-up as facilitators of management CoC [14, 15, 18, 19, 29, 31]. Patients reported positive [30] or negative impacts on management CoC [3].

### Informational CoC

Two quantitative studies reported on the informational domain of CoC: In the study by Uijen, patients in all three care modes felt that their care providers knew them and communicated well, independent of the number of care providers seen [26]. Tourigny also found an increase in the informational CoC sub score using a modified version of the PCAT [22].

Qualitative studies were more mixed: In the studies by Abelson and Norful, nurses in the team were seen as being well informed on patient issues and as enablers of informational CoC [14, 30]. However, HCPs and patients in several studies mentioned that delegation in team-based care may act as a barrier to informational CoC as it could lead to a loss of overview with decreasing direct involvement in patient care [27] and that involving multiple providers one patients care may result in miscommunication and conflicting medical advice [19, 31].

### Management CoC

Two quantitative studies mentioned management CoC. Uijen et al. showed smooth and connected care in all three care modes (i.e. no differences in the sub scores relating to management CoC of the team CoC questionnaire). This was independent of the number of care providers seen [26]. The study by Duhoux used a modified PCAT tool reporting a single CoC score, that encompassed both the relational and management domain, and which showed variable scores and very little change over time in all IPCTs. The study did not report a separate score for each domain.

Qualitative studies provided more information about this domain [14, 15, 18, 19, 29, 31]:While HCPs mostly found that involving new professionals in coordinative tasks and patient follow-up improved management CoC [14, 15, 18, 19, 29, 31], not all patients agreed. Whilst the patients in the study by Abelsen et al. [30] found their care to be better coordinated, patients in the study by McDermott et al. [3] found follow up appointments with the same practitioner to be more complicated to obtain.

### Relational CoC

This domain was the most extensively studied by the papers included in this review. One of the most common tools used to measure relational CoC with a preferred or usual provider is the UPC. The unmodified UPC measures only the proportion of visits to a usual PCP, and it therefore unsurprisingly declined in two of the three studies using it after skill-mix interventions were introduced. Both of those studies, however, reported improved CoC when using alternative measures (patient-reported team CoC [26] or PCAT [22]). The study by Lewis et al. reports an increased UPC after expanding multiprofessional care and is therefore particularly interesting [16]. This may be connected to certain measures introduced by the intervention in the study, such as the “My RN” concept, which assigned an RN to each patient as is traditional with PCPs. This ensured each patient would know his/her nurse and the nurse would know each patient.

The three large scale surveys included in this review focused on relational CoC, with all three finding poorer relational CoC scores in larger, team-based primary care settings compared to smaller, GP-led practices [20, 28, 33].

All versions of the PCAT employed by studies included the relational domain of CoC and showed mixed outcomes. Two studies indicated a decrease in PCAT-CoC either after the introduction of collaborative teams into community-based family practices [21] or with an increased number of providers and nurses in practices [24]. One study found very little change [23] and one found increased PCAT scores [22]. Both studies indicating increased or unchanged PCAT scores had increased their multiprofessional involvement through reforms encompassing greater changes than solely introducing new professionals. These changes included measures specifically designed to maintain CoC [19, 20].

The majority of qualitative studies focused on the relational domain of CoC. Patients and HCPs both suggested that PCPs could improve their availability by delegating certain tasks which may improve relational CoC [14, 15, 27, 32]. Quantitative findings did not necessarily support this. As described above, the UPC, which most directly captures face-to-face CoC with a PCP, was lower in two out of three studies employing it [22, 26] and higher in one study [16]. Two of the large scale surveys [20, 28] had patients estimate the percent of visits to the usual PCP. Both studies found negative association with multidisciplinary practice teams. As mentioned above, HCPs were not unilaterally positive towards delegation, suggesting it may come at the cost of informational CoC.

Patients’ and providers’ perspectives appear divergent on relational CoC. In the view of HCPs relational CoC is provided by the entire team [25, 27] including non-PCP team members [15, 29]. In one study, patients agreed that nurses strengthened their link to their PCP [30]. Nevertheless, in most studies patients highlighted the importance of maintaining close connection to their usual PCP [3, 27, 32] or had concerns about disrupting long established relationships [3]. This divergence echoes findings from a Cochrane review evaluating nurse-doctor substitution in a larger setting. This review reported that in the perception of PCPs, doctor-nurse substitution improved CoC while recipients of care in some studies were concerned over the CoC provided by nurses and felt insecure if they lost contact with their PCPs.

The idea of effective team-based care often relies on the assumption that the absence of relational CoC with a single provider can be substituted by strong informational CoC within the team, for example by clinicians having access to shared patient records, and electronic messaging. However, a recent case study on the subject [37] showed that this assumption does not always hold true. The potential for continuous care was found to be even more dependent on staff, patient, and system factors such as the attitude of clinicians to care coordination and the ability of an organization to retain staff. This sentiment was shared by patients in the study by Groot et al., where patients acknowledged that tools such as an electronic health record may help support informational CoC, but felt that it was of less value than having a strong personal relationship with a PCP [27]. Further, when multiple health professionals are involved in data entry, this may also increase the potential risk of input errors.

### Differences by team size and composition

HCPs emphasized that small, stable teams and strong communication were key facilitators of CoC [27, 31]. This aligns with quantitative findings by Huang et al., where mid-sized teams of three to four physicians and NP/PAs achieved the highest CoC levels [17]. Although large teams may increase availability and the number of visits with a care team provider, this may come at the expense of relational CoC. Large practice size was consistently associated with lower CoC scores across the large-scale surveys [20, 28, 33]. Co-location was highlighted as beneficial [18, 19], as this allows clinicians to share information effectively and enables patients to receive all care from a single practice. These findings of CoC being likely to suffer in large teams is shared by a 2025 scoping review evaluating the effects of multidisciplinary teams in primary care, which highlighted negative effects especially on the relational domain [38]. Addressing these challenges, teamlets or micro-teams were built. They may take responsibility for a defined patient population within a bigger multidisciplinary team which leads to reduced numbers of contact for patients. Coombs et al.’s review [39] found that micro-teams are often conceptualized flexibly in practice and moderate losses to CoC in expanding practices. They emphasized the distinction between CoC with an individual clinician and CoC provided by a team, highlighting that effective and sustained communication is essential to achieve the latter.

Regarding team composition, the implementation of skill-mix remains a relatively novel challenge in many countries. Nursing played a central role in our findings, as every study included in this review involved nursing professionals. This may be linked to the current interest in expanding advanced nursing roles within primary care practices in some countries. For example, APNs are still new to many patients. In this review, we showed that patients may not yet fully understand these new roles [3, 27]. With time and acceptance, nurse practitioners contributed to CoC [3].

Furthermore, expansion of skill-mix was perceived as reshaping the role of some PCPs. Non-PCP practitioners tended to manage less complex cases, while PCPs increasingly focused on patients with more complex needs. Consequently, PCPs reported their work was becoming more comparable to that of a hospital consultant [3].

### Differences by research method and measurement tool

Our findings were strongly linked to the research method used as well as the context of each study. For example, interventional studies examining large-scale health care reforms tended to show positive effects [16, 22, 25, 26, 29, 30], whereas cross-sectional or descriptive studies focusing solely on correlations between the presence of multiple professionals and CoC were more likely to report negative findings [3, 14, 19, 20, 24, 27, 28]. This might indicate that role distribution in CoC is complex, and that conventional quantitative measures may not fully capture the domains of “CoC” experienced by patients.

The PCAT for example was developed over 20 years ago, and the definition of CoC used to develop it may no longer be conform to a modern understanding of the concept. Many influential frameworks on CoC were developed around or after the tool was released. The landmark paper by Haggerty, now one of the most widely used frameworks, was published 2 years after the PCAT. Maybe informatively, the two studies reporting a decreased score used an unmodified version of the tool, whereas the two other studies used a modified one.

Commonly used indices often fail to capture relevant outcomes in team-based care settings. For example, the UPC will always decrease when a patient is seen by more than one professional or provider, no matter how connected the patients care is. This raises questions about the usefulness of the UPC in team-based settings. Validated instruments have already been developed for such problems. One such tool was described by Haggerty et al. in 2012 [8]. However, none of the included studies used these instruments. This may be due to limited awareness or adoption. Another assessment tool for evaluation of the quality of team-based care is visit entropy (VE) [40, 41]. Visit entropy quantifies the amount of unpredictability or disorganization in a patient’s previous visit pattern to clinicians. Although team-based multidisciplinary approaches may decrease traditional CoC metrics, VE could capture improvements in care delivery, as highly functional, multiprofessional teams may provide a more organized and predictable care experience than other forms of care.

### Strengths and limitations

As a strength, This scoping review rigorously followed the guidelines of the PRISMA-ScR checklist [42]. A thorough search strategy was designed, and four databases were searched. One limitation, or rather a challenge of this study is the wide-ranging understanding of what the concept of CoC comprises. During the search we based our understanding and definition on the framework developed by Haggerty [5]. This approach allowed the inclusion of studies that examined CoC-related outcomes even if they did not explicitly employ the term “CoC,” and thereby broadened the scope of our search. While the Haggerty framework provided a structured approach it is possible that some relevant studies were missed or interpreted differently depending on their conceptualization of CoC. Secondly, because many studies implemented multiple interventions at once, in some cases it is difficult to know whether the effects are really due to team-based care.

Overall, study quality was generally acceptable to strong, particularly among quantitative studies, although methodological heterogeneity was evident in terms of study design, sample size, and operationalisation of CoC. While most studies were methodologically sound within their respective designs, this makes drawing overarching conclusions challenging when comparing studies and should be considered when interpreting the synthesis. The methodological quality of the included studies was not formally appraised, consistent with scoping review methodology.

A scoping review methodology was used to map the extent, range, and nature of evidence on multiprofessional primary care and continuity of care. This design is particularly suited to research areas characterized by heterogeneous study designs and methodologies and encompasses the identification of key concepts, sources, and the types of evidence available. In doing so scoping reviews may contribute to a comprehensive understanding of the evidence landscape and identify research gaps, laying the foundation for informed research priority-setting [43]. We are confident that this scoping review will provide a valuable overview and baseline for future research and systematic reviews.

### Implications regarding CoC

The results imply that multiprofessional care within the primary care practice may come at the cost of CoC. However, factors beyond team composition may exert an equal or greater influence on whether CoC is preserved or lost. These factors include practice organization and the manner in which new professional roles are introduced, including whether reforms incorporate measures intended to preserve CoC. A small practice size, stable teams and good quality of communication in the team were consistently mentioned as key facilitators of CoC. Across studies, relational CoC was the most negatively affected whereas informational and management CoC were generally maintained or even improved. Finally, many quantitative measures such as the UPC fail in team-based settings and may not fully capture patients lived experiences. Future research should employ measurement tools specifically developed to measure CoC in multiprofessional context. PCPs should be aware that maintaining a high level of relational CoC is precarious while introducing new professional roles in their practice and that special efforts to strengthen relational CoC are needed.

## Conclusion

The relationship between continuity of care and multiprofessional care is complex and context dependent. When new professional roles are introduced in primary care practice teams, multiprofessional care may offer the opportunity to maintain or even improve continuity of care. Importantly, expectations and concerns of providers and patients must be considered and addressed, especially for relational continuity of care which is a major concern for patients in the context of multiprofessional care.

## Supplementary Information

Supplementary Material 1.

Supplementary Material 2.

Supplementary Material 3.

## Data Availability

All data generated or analysed during this study are included in this published article and its supplementary information files.
