# Supplementary material for: Impact of multiprofessional collaboration in primary care practices on continuity of care: a scoping review
Source: BMC Prim Care. 2026 Aug 3;27:395. doi: 10.1186/s12875-026-03507-y (PMC13721453; doi:10.1186/s12875-026-03507-y)
Supplement: Supplementary file 1 [file 12875_2026_3507_MOESM2_ESM.pdf]

EBSCO	PubMed	Cochrane Library	EMBASE
(<br>MH "Interprofessional Relations"<br>OR MH "Nurse-Physician Relations"<br>OR MH "Nurse Practitioners"<br>OR MH "Multidisciplinary Care Team"<br>OR TI (<br>nurse-practitioner*<br>OR advanced practice nurs*<br>OR nurse-clinician*<br>OR co-location<br>OR interprofession*<br>OR inter-profession*<br>OR interdisciplin*<br>OR inter-disciplin*<br>OR multiprofession*<br>OR multi-profession*<br>OR crossdisciplinar*<br>OR cross-disciplinar*<br>OR transdisciplin*<br>OR trans-disciplin*<br>OR team-based care<br>OR skill-mix<br>OR advanced nursing practice<br>OR nursing role*<br>OR nursing team*<br>OR nurse*<br>OR multidisciplin*<br>OR multi-disciplin*<br>)<br>OR AB (<br>nurse-practitioner*<br>OR advanced practice nurs*<br>OR nurse-clinician*<br>OR co-location<br>OR interprofession*<br>OR inter-profession*<br>OR interdisciplin*<br>OR inter-disciplin*<br>OR multiprofession*<br>OR multi-profession*<br>OR crossdisciplinar*<br>OR cross-disciplinar*<br>OR transdisciplin*<br>OR trans-disciplin*<br>OR team-based care<br>OR skill-mix<br>OR advanced nursing practice<br>OR nursing role*<br>OR nursing team*<br>OR nurse*<br>OR multidisciplin*<br>OR multi-disciplin*<br>)<br>)<br><br>AND<br>(<br>MH "Physicians, Family"<br>OR MH "Family Practice"<br>OR MH "Primary Health Care"<br>OR TI (<br>general practitioner*<br>OR GP<br>OR GPs<br>OR family medicine<br>OR family physician*<br>OR family practitioner*<br>OR family practice<br>OR general practice physician*<br>OR primary care practitioner*<br>OR primary health care<br>OR primary care<br>OR general practice<br>OR generalist physician*<br>)<br>OR AB (<br>general practitioner*<br>OR GP<br>OR GPs<br>OR family medicine<br>OR family physician*<br>OR family practitioner*<br>OR family practice<br>OR general practice physician*<br>OR primary care practitioner*<br>OR primary health care<br>OR primary care<br>OR general practice<br>OR generalist physician*<br>)<br>)<br><br>AND<br>(<br>MH "Continuity of Patient Care"<br>OR TI (<br>continuity<br>OR continuity of care<br>OR continuum of care<br>OR continued care<br>OR continuous care<br>OR continuity of healthcare<br>OR care continuity<br>OR continuity of patient care<br>OR connected care<br>OR ongoing care<br>OR seamless care<br>OR coordinated care<br>OR co-ordinated care<br>OR patient-centered care<br>OR patient-centred care<br>OR patient focused care<br>OR continuing care<br>OR fragmentation<br>OR discontinuity<br>OR patient perception*<br>OR perception* of patients<br>OR patient perceived<br>OR patient experience*<br>OR healthcare experience*<br>OR patient care quality<br>OR quality of patient care<br>OR integration of care<br>OR integration of healthcare<br>OR integrated care<br>)<br>OR AB (<br>continuity<br>OR continuity of care<br>OR continuum of care<br>OR continued care<br>OR continuous care<br>OR continuity of healthcare<br>OR care continuity<br>OR continuity of patient care<br>OR connected care<br>OR ongoing care<br>OR seamless care<br>OR coordinated care<br>OR co-ordinated care<br>OR patient-centered care<br>OR patient-centred care<br>OR patient focused care<br>OR continuing care<br>OR fragmentation<br>OR discontinuity<br>OR patient perception*<br>OR perception* of patients<br>OR patient perceived<br>OR patient experience*<br>OR healthcare experience*<br>OR patient care quality<br>OR quality of patient care<br>OR integration of care<br>OR integration of healthcare<br>OR integrated care<br>)<br>)<br><br>NOT<br>(<br>TI (<br>autobiography<br>OR biography<br>OR comment<br>OR conference<br>OR editorial<br>OR lecture<br>OR newspaper article<br>OR personal narrative<br>)<br>OR AB (<br>autobiography<br>OR biography<br>OR comment<br>OR conference<br>OR editorial<br>OR lecture<br>OR newspaper article<br>OR personal narrative<br>)<br>)	(<br>"Interprofessional Relations" [Mesh]<br>OR "Physician-Nurse Relations" [Mesh]<br>OR "Nurse Practitioners" [Mesh]<br><br>OR nurse-practitioner* [TIAB]<br><br>OR advanced practice nurse* [TIAB]<br><br>OR nurse-clinician* [TIAB]<br>OR co-location [TIAB]<br><br>OR interprofession* [TIAB]<br>OR inter-profession* [TIAB]<br>OR interdisciplin* [TIAB]<br>OR inter-disciplin* [TIAB]<br>OR multiprofession* [TIAB]<br>OR multi-profession* [TIAB]<br>OR crossdisciplinar* [TIAB]<br>OR cross-disciplinar* [TIAB]<br>OR transdisciplin* [TIAB]<br>OR trans-disciplin* [TIAB]<br>OR team-based care [TIAB]<br>OR skill-mix [TIAB]<br>OR advanced nursing practice [TIAB]<br>OR nursing role* [TIAB]<br>OR nursing team* [TIAB]<br>OR nurse* [TIAB]<br>OR multidisciplin* [TIAB]<br>OR multi-disciplin* [TIAB]<br>)<br><br>AND<br>(<br>"Physicians, Primary Care" [Mesh]<br>OR "General Practice" [Mesh]<br><br>OR general practitioner* [TIAB]<br>OR GP [TIAB]<br>OR GPs [TIAB]<br>OR family medicine [TIAB]<br>OR family physician* [TIAB]<br>OR family practitioner* [TIAB]<br>OR family practice [TIAB]<br>OR general practice physician* [TIAB]<br>OR primary care practitioner* [TIAB]<br>OR primary health care [TIAB]<br>OR primary care [TIAB]<br>OR general practice [TIAB]<br>OR generalist physician* [TIAB]<br>)<br><br>AND<br>(<br>"Continuity of Patient Care" [Mesh]<br>OR continuity [TIAB]<br>OR continuity of care [TIAB]<br>OR continuum of care [TIAB]<br>OR continued care [TIAB]<br>OR continuous care [TIAB]<br>OR continuity of healthcare [TIAB]<br>OR care continuity [TIAB]<br>OR continuity of patient care [TIAB]<br>OR connected care [TIAB]<br>OR ongoing care [TIAB]<br>OR seamless care [TIAB]<br>OR coordinated care [TIAB]<br>OR co-ordinated care [TIAB]<br>OR patient-centered care [TIAB]<br>OR patient-centred care [TIAB]<br>OR patient focused care [TIAB]<br>OR continuing care [TIAB]<br>OR fragmentation [TIAB]<br>OR discontinuity [TIAB]<br>OR patient perception* [TIAB]<br>OR perception* of patients [TIAB]<br>OR patient perceived [TIAB]<br>OR patient experience* [TIAB]<br>OR healthcare experience* [TIAB]<br>OR patient care quality [TIAB]<br>OR quality of patient care [TIAB]<br>OR integration of care [TIAB]<br>OR integration of healthcare [TIAB]<br>OR integrated care [TIAB]<br>)<br><br>NOT<br>(<br>autobiography [TIAB]<br>OR biography [TIAB]<br>OR comment [TIAB]<br>OR conference [TIAB]<br>OR editorial [TIAB]<br>OR lecture [TIAB]<br>OR newspaper article [TIAB]<br>OR personal narrative [TIAB]<br>)	(<br>(<br><br>"Interprofessional Relations"<br>OR "Nurse-Physician Relations"<br>OR "Multidisciplinary Care Team"<br>OR "nurse practitioner"<br>OR "Nurse Practitioners"<br>OR "advanced practice nurse"<br>OR "advanced practice nurses"<br>OR "nurse-clinician"<br>OR "nurse-clinicians"<br>OR "co-location"<br>OR interprofession*<br>OR inter-profession*<br>OR interdisciplin*<br>OR inter-disciplin*<br>OR multiprofession*<br>OR multi-profession*<br>OR crossdisciplinar*<br>OR cross-disciplinar*<br>OR transdisciplin*<br>OR trans-disciplin*<br>OR "team-based care"<br>OR "skill-mix"<br>OR "advanced nursing practice"<br>OR "nursing role"<br>OR "nursing team"<br>OR nurse*<br>OR multidisciplin*<br>OR multi-disciplin*<br><br>)<br><br>AND<br>(<br>"Primary Health Care"<br>OR "general practitioner"<br>OR "general practitioners"<br>OR "GP"<br>OR "GPs"<br>OR "family medicine"<br>OR "family physician"<br>OR "family physicians"<br>OR "family practitioner"<br>OR "family practitioners"<br>OR "Family Practice"<br>OR "general practice physician"<br>OR "general practice physicians"<br>OR "primary care practitioner"<br>OR "primary care practitioners"<br>OR "primary care"<br>OR "general practice"<br>OR "generalist physician"<br>OR "generalist physicians"<br><br>)<br><br>AND<br>(<br><br>continuity<br>OR "continuity of care"<br>OR "continuum of care"<br>OR "continued care"<br>OR "continuous care"<br>OR "continuity of healthcare"<br>OR "care continuity"<br>OR "continuity of patient care"<br>OR "connected care"<br>OR "ongoing care"<br>OR "seamless care"<br>OR "coordinated care"<br>OR "co-ordinated care"<br>OR "patient-centered care"<br>OR "patient-centred care"<br>OR "patient focused care"<br>OR "continuing care"<br>OR "fragmentation"<br>OR "discontinuity"<br>OR "patient perception"<br>OR "patient perceptions"<br>OR "perceptions of patients"<br>OR "patient perceived"<br>OR "patient experience"<br>OR "patient experiences"<br>OR "healthcare experience"<br>OR "patient care quality"<br>OR "quality of patient care"<br>OR "integration of care"<br>OR "integration of healthcare"<br>OR "integrated care"<br><br>)<br>):ti,ab,kw	(<br>'doctor nurse relation'/exp<br>OR 'collaborative care team'/exp<br>OR 'advanced practice provider'/exp<br>OR 'skill mix'/exp<br>OR 'teamwork'/exp<br><br>OR 'nurse-practitioner*':ti,ab,kw<br>OR 'advanced practice nurse*':ti,ab,kw<br>OR 'nurse-clinician*':ti,ab,kw<br>OR 'co-location':ti,ab,kw<br>OR 'interprofession*':ti,ab,kw<br>OR 'inter-profession*':ti,ab,kw<br>OR 'interdisciplin*':ti,ab,kw<br>OR 'inter-disciplin*':ti,ab,kw<br>OR 'multiprofession*':ti,ab,kw<br>OR 'multi-profession*':ti,ab,kw<br>OR 'crossdisciplinar*':ti,ab,kw<br>OR 'cross-disciplinar*':ti,ab,kw<br>OR 'transdisciplin*':ti,ab,kw<br>OR 'trans-disciplin*':ti,ab,kw<br>OR 'team-based care':ti,ab,kw<br>OR 'skill-mix':ti,ab,kw<br>OR 'advanced nursing practice':ti,ab,kw<br>OR 'nursing role*':ti,ab,kw<br>OR 'nursing team*':ti,ab,kw<br>OR 'nurse*':ti,ab,kw<br>OR 'multidisciplin*':ti,ab,kw<br>OR 'multi-disciplin*':ti,ab,kw<br>)<br><br>AND<br>(<br>'general practitioner'/exp<br>OR 'general practice'/exp<br>OR 'primary health care'/exp<br>OR 'general practitioner*':ti,ab,kw<br>OR 'gp':ti,ab,kw<br>OR 'gps':ti,ab,kw<br>OR 'family medicine':ti,ab,kw<br>OR 'family physician*':ti,ab,kw<br>OR 'family practitioner*':ti,ab,kw<br>OR 'family practice':ti,ab,kw<br>OR 'general practice physician*':ti,ab,kw<br>OR 'primary care practitioner*':ti,ab,kw<br>OR 'primary health care':ti,ab,kw<br>OR 'primary care':ti,ab,kw<br>OR 'general practice':ti,ab,kw<br>OR 'generalist physician*':ti,ab,kw<br>)<br>AND<br>(<br>'Person centered care'/exp<br>OR 'continuity':ti,ab,kw<br>OR 'continuity of care':ti,ab,kw<br>OR 'continuum of care':ti,ab,kw<br>OR 'continued care':ti,ab,kw<br>OR 'continuous care':ti,ab,kw<br>OR 'continuity of healthcare':ti,ab,kw<br>OR 'care continuity':ti,ab,kw<br>OR 'continuity of patient care':ti,ab,kw<br>OR 'connected care':ti,ab,kw<br>OR 'ongoing care':ti,ab,kw<br>OR 'seamless care':ti,ab,kw<br>OR 'coordinated care':ti,ab,kw<br>OR 'co-ordinated care':ti,ab,kw<br>OR 'patient-centered care':ti,ab,kw<br>OR 'patient-centred care':ti,ab,kw<br>OR 'patient focused care':ti,ab,kw<br>OR 'continuing care':ti,ab,kw<br>OR 'fragmentation':ti,ab,kw<br>OR 'discontinuity':ti,ab,kw<br>OR 'patient perception*':ti,ab,kw<br>OR 'perception* of patients':ti,ab,kw<br>OR 'patient perceived':ti,ab,kw<br>OR 'patient experience*':ti,ab,kw<br>OR 'healthcare experience*':ti,ab,kw<br>OR 'patient care quality':ti,ab,kw<br>OR 'quality of patient care':ti,ab,kw<br>OR 'integration of care':ti,ab,kw<br>OR 'integration of healthcare':ti,ab,kw<br>OR 'integrated care':ti,ab,kw<br>)<br>NOT (<br>'autobiography':ti,ab,kw<br>OR 'biography':ti,ab,kw<br>OR 'comment':ti,ab,kw<br>OR 'conference':ti,ab,kw<br>OR 'editorial':ti,ab,kw<br>OR 'lecture':ti,ab,kw<br>OR 'newspaper article':ti,ab,kw<br>OR 'personal narrative':ti,ab,kw<br>)<br><br>AND (([embase]/lim OR [preprint]/lim) NOT ([embase]/lim AND [medline]/lim)) NOT 'conference abstract'/it
