# Supplementary material for: Impact of multiprofessional collaboration in primary care practices on continuity of care: a scoping review
Source: BMC Prim Care. 2026 Aug 3;27:395. doi: 10.1186/s12875-026-03507-y (PMC13721453; doi:10.1186/s12875-026-03507-y)
Supplement: Supplementary file 3 [file 12875_2026_3507_MOESM1_ESM.docx]

| **Title** | **Authors and year** | **Location and Setting** | **Study design** | **Population** | **Professions discussed in study** | **Aim and Intervention** | **Impact of multi-professional co-location on continuity** | **Measures and frameworks of continuity used** |
| --- | --- | --- | --- | --- | --- | --- | --- | --- |
| **Continuity in different care modes and its relationship to quality of life: a randomised controlled trial in patients with COPD.** | Uijen AA, 2012 | 15 general practices from the Nijmegen region, the Netherlands | Single-blinded, parallel-group, randomised controlled trial with 2-year follow-up | 180 patients with COPD | PCP  Practice Nurse | Aim: ”To analyse whether experienced continuity for patients with chronic obstructive pulmonary disease (COPD) changes after different care modes are introduced, and to analyse the relationship between continuity of care and patients ’quality of life.”  Intervention: ”Patients were randomly  assigned to three different care modes: self-  management, regular monitoring by a practice  nurse, and care provided by the GP at the  patient’s own initiative (usual care).” | 1) ”Personal continuity was significantly higher for patients receiving usual care compared with patients in the other care modes”:   - Usual Care: 0.74 (95% CI: 0.61 to 0.86) - Self management: 0.36 (95% CI: 0.25 to 0.47) (P= 0.00) - Regular monitoring by practice nurse: 0.50 (95% CI: 0.39 to 0.61) (P= 0.02)   2) No differences in experienced team continuity in primary care among the three care modes were found between baseline and 2 years:   - Usual Care: –0.05 (95% CI: –1.84 to 1.74) - Self management: –0.21 (95% CI: –1.66 to 1.25) (P= 0.883) - Regular monitoring by practice nurse: 0.12 (95% CI: –1.43 to 1.68) (P= 0.873) | 1. Personal continuity was measured using the Usual Provider of Continuity (UPC) index (range: 0-1) 2. Team continuity was measured using a questionnaire including six items on team continuity. A total score of team continuity was calculated (range: 6-30) |
| **Expanding general practice with interprofessional teams: a mixed‑methods patient perspective study** | Abelsen B, 2023 | 17 general practices in Norway | Mixed-methods approach using interviews and quantitative information collected in a survey | Interviews: 10 patients, 4  next of kin  Survey: 287 respondents  Each practice included between 3 and 17 regular GPs, 1-4 nurses, and 2-6 medical assistants | PCP  Nurse | Aim: ”This study aims to explore patients’ experiences when their usual primary healthcare encounter with general practice shifts from single-profession general practitioner care to interprofessional team-based care.”  Intervention: "The pilot expanded general practices with nurses and  a [primary healthcare team] includes regular GPs, nurses, and medical assistants" | Patients perceived all three types of continuity (informational, relational and management continuity) as strengthened when their primary healthcare encounter with general practice transitioned from single-profession GP care to interprofessional team-based care.  The patient interview and survey reported follow-ups in team-based care to be characterized by a greater degree of planning in patient treatment, more personally fitted, better coordinated and offering increased consultation time. Patients also reported increased understanding and mastering of  patients’ health problems. The study labeled this as improved management continuity.  Informational continuity was also reported to be improved, especially by multi-morbid elderly patients and interviewees with mental illness, who found that important concerns they had raised with the nurse were known to the GP and that they did not have to repeat themselves.  Relationship continuity was also reported as strengthened: Respondents experienced continuity in their relationship with the nurse and several interviewees talked about the nurse as strengthening the link between them and the GP. None of the interviewees expressed dissatisfaction with the inclusion of a nurse in their general practitioner relationship. | Informational, relational and management continuity as described by Haggerty et al.  [Haggerty J, Reid R, Freeman G, Starfield B, Adair C, McKendry R. Conti-  nuity of care: a multidisciplinary review. BMJ. 2003;327:1219–21.] |
| **Improving personal continuity in general practice: a focus group study.** | Groot L, 2024 | 4 focus groups, Netherlands | Qualitative study using 4 focus groups | 17 GPs, 7 patients, 4 practice assistants, 2 practice nurses | PCP  Practice Nurse  Practice Assistant | Aim: ”To explore the perspectives of GPs, older patients, practice nurses, and assistants on improving personal continuity in general practice, and to identify barriers and facilitators that affect this improvement process.” | In a team-based setting ”GPs, practice assistants, and practice nurses viewed personal continuity as being provided by the entire general practice team and not just by the patient’s own GP”. GPs, practice assistants, and practice nurses viewed having a personal GP only necessary for certain patient groups, mainly patients with complex chronic diseases. However, Patients often viewed having their own GP as central in their concept of personal continuity.  It was suggested that continuity for these groups could be improved by retaining the availability and accessibility of the patient’s own GP by delegating certain patient-related tasks tasks to practice nurses and administrative tasks to a practice manager. However, practice assistants and nurses found patients often were not familiar with their roles and were therefore reluctant to accept their role. GPs also mentioned delegating tasks may lead to a loss of overview with decreasing direct involvement in patient care. GPs, practice assistants, and practice nurses found having small, stable teams with low staff turnover and improving team communication (for example by efficient use of electronic health records) to be beneficial to personal continuity. Patients also acknowledged the value of their EHR, yet they felt that it was of less value than having personal contact. | Personal continuity, team continuity and cross-boundary continuity as defined by Uijen et al. [Uijen AA, Schers HJ, Schellevis FG, et al.  How unique is continuity of care? A review  of continuity and related concepts. Fam  Pract 2012; 29(3): 264–271.] |
| **Interprofessional care of patients with type 2 diabetes mellitus in primary care: family physicians' perspectives.** | Torti J, 2022 | Family practices affiliated with one primary care network (PCN) in Edmonton, Alberta, Canada. | Qualitative study using interviews with family physicians | 15 family physicians | PCP  Nurse  Dietitian  Pharmacist  ”Other allied health professionals” | Aim: ”The purpose of this study was to explore  physicians’ perspectives of barriers and facilitators to  interprofessional team-based care of patients with  T2DM within family practice settings.” | It was found that ”having many providers involved in the care of a patient with T2DM was  perceived to be a barrier to patient care by jeopardizing continuity of care.” This was attributed to that involving multiple providers one patients care may result in miscommunication and conflicting medical advice.  However: ”Co-location, as facilitated by a decentralized PCN model,  enabled physicians to share physical space with other  health professionals, which helped to forge relationships  and allowed for face-to-face communication. When  team members were on-site and readily available, patient  appointments were better coordinated with various PCN  health professionals.” This may be interpreted as management continuity or informational continuity. | Not specified |
| **Practice list size, workforce composition and performance in English general practice: a latent profile analysis.** | Kayira A, 2024 | 6024 general practices, England, UK | Latent profile analysis of five English practice level datasets (General Practice workforce, General Practice Patient Survey, NHS Payments to General Practice, Quality and Outcomes Framework (QOF) and National General Practice Profiles) | Data from 6024 general practices | PCP  Nurses  Paramedics Pharmacist  Health care assistant (HCA)  Administrative staff  ”Other allied health professionals (AHP)” | Aim: ”We hypothesised that there exists distinct patterns of practice list size and workforce composition which may be associated with practice performance. We sought to identify these practice profiles or subgroups and assess whether membership to a particular group determined how a GP practice performed in primary care indicators.” | Practices were sorted into 3 groups based on list size and workforce composition: 1) Small practices more reliant on GPs 2) medium-size GP-led practices with a multidisciplinary team (MDT) input and 3) large multidisciplinary practices.  ”Continuity of care was 20.9% poorer (32.6% vs. 41.2%) for medium size practices with MDT input and 34.5% worse (27.0% vs. 41.2%) for large multidisciplinary practices compared to small GP dependent practices (p < 0.001). ” | Continuity was measured as the percentage of patients reporting to see or speak to their preferred GP always, almost always or a lot of the time in the survey analyzed |
| **Predictors of relational continuity in primary care: patient, provider and practice factors.** | Kristjansson E, 2013 | 137 practices in Ontario, Canada | Patient, provider and practice survey | 363 health  practitioners, 5295 patients | PCP  Nurse | Aim: ”Our purpose was to assess predictors of continuity of care within a large sample of primary care practices in Ontario, Canada. A secondary question assessed whether there was a difference between organizational models of primary care in the continuity of care provided.” | The study found that having more nurses in a practice was related to lower continuity, irrespective of practice size:  ”The continuity score decreased with the increase in the number of providers (effect size =−0.33%); it also decreased with the number of nurses (effect size =−0.64%).” | Four-items from the Primary Care Assessment tool included in the patient survey measured relational continuity and were used to calculate a score ranging from .25 to 1.0 with  higher scores reflecting greater perceived continuity.  [Shi L, Starfield B, Xu J: Validating the adult primary care assessment tool.  J Fam Pract 2001, 50:161.] |
| **The benefits of co-location in primary care practices: the perspectives of general practitioners and patients in 34 countries.** | Bonciani M, 2018 | 31 European Countries and 3 non-European (Australia, Canada and New Zealand) | Questionnaire-based cross-sectional survey, using data from QUALICO-PC study | 7183 GPs, 61931 Patients | PCP  Nurse  Midwife Physiotherapist Dentist  Pharmacist  Social worker | Aim: ”The aim of  this study was to identify the relationships between general practitioner (GP) co-location with other GPs and/or  other professionals and the GP outcomes and patients’ experiences.” | ”Compared to patients of GPs working in single-handed practices, patients perceive less continuity of care when their GPs are co-located only with other professionals or with other GPs and other professionals (p < 0.01, […]). This negative association with continuity of care is also present as one of the main effects / as the main effect of GP co-location in a multi-professional setting (p < 0.01, […]), and in practices where GPs are co-located with nurses, patients  experience even less continuity of care (p < 0.001, […]).” | 3 questions in the patient questionnaire on whether GPs have known  the patient on a long-term basis |
| **The perspectives of nurse practitioners and physicians on increasing the number of registered nurses in primary care.** | Norful A, 2018 | United States | Qualitative descriptive design using interviews with primary care providers | 12 physicians and 14 nurse-practitioners, from various Primary Care Practices in New York State including physician-owned practices (n=5), university-  affiliated clinics (n=9), hospital-affiliated clinics (n=11), and a federally qualified health  center (n=1). | PCP  Nurse practitioners | Aim: ”The purpose of this study is to elicit PCP [primary care providers] perspectives about the use of RNs in primary care, including how RNs can optimally be integrated into primary care teams and the perceived impact of increased RN staffing in primary care.” | Almost all PCPs interviewed perceived an increase in continuity of care when registered nurses (RNs ) were incorporated into primary-care teams especially as the volume of patients and complexity of care needs increases. RNs help to ensure  that no important care management tasks are left undone or omitted. RNs were perceived as being well informed of individual patients issues and involved in patient education and shared decision making. Patient follow-up, either by telephone or in-person, and co-management of patients by RNs was perceived to enhance continuity. PCPs also reported that delegating tasks to RNs helped to reduce PCP workload and the strain to complete all care management task singlehandedly, thereby freeing up time for additional patient encounters and administrative tasks. | Not specified |
| **Physician Use of Health Care Teams for Improving Quality in Primary Care.** | Hoff T, 2019 | Different primary care settings in the northeastern region of the United States. | Qualitative study using data collected through interviews | 39 primary care physicians and 9 medical assistants | PCP  Nurse  Medical Assistant | Aim: ”This study’s aim was to explore how physicians use teams in practice to deliver higher-quality  care.” | Physicians found team-based care to improve continuity by 1) “getting basic care duties off their  plate” and 2) team members acting as “as relational extensions” of the physicians to enhance the  patient experience and provide care continuity.  Other team members (ie, nurses, MAs, care managers, social workers) were described as being able to perform basic primary care work thereby creating additional time capacity for providers to perform the complex aspects of their work with patients. Physicians mentioned this enhanced the doctor patient relationship.  Team members were said to act as a relational liaison, extending the “voice” of the physician with patients, enhancing relational continuity by making the team an extension of the physician.  It was also reported that team members enabled physicians to get to know the patient better. Medical assistants were described as providing a different perspective from the physician and gathering additional information about the patient.  Finally team members were also reported as being involved in Complex care coordination. | Not specified |
| **A model for patient-centered Army primary care.** | Lewis P, 2012 | 1 military family practice-based setting, within a  military community hospital, United States | Pilot program, non-randomized, using administrative data | - Pilot program: Approximately 2,500 empanelled patients, 2 PCPs (primary care providers: 1 family practice physician, 1 physician assistant), and support staff (1 Registered Nurse (RN), 2 RN case managers, 2 Licensed practical Nurses, 1 certified nursing assistant, and 1 data assistant) functioning as 2 teamlets. - Comparison group: Panel size: 1127.7 patients per Provider and 12 PCPs (family practice physicians, family nurse practitioners, and physician assistants) | PCP  Physician assistant  Registered Nurse  Registered nurse case manager Licensed practical nurse  Certified nursing assistant  Data assistant | Aim: The study evaluated novel method of primary care delivery and compared it to usual care: ”This pilot specifically sought to address expanded nursing roles, population management, demand management, and increased access to care.”  Intervention: A PCP, a RN, and a RN case manager formed a teamlet all equally responsive and responsible for the empanelled patient population. The model introduced the “My RN” concept, which assigned an RN to each patient as is traditional with providers. This ensured each patient would know his/her nurse and the nurse would know each patient. | The project reported an increased continuity of care in the pilot group. The pilot group achieved a significantly higher score of 72.7% (p = 0.006) in the measure of ”Provider’s Ability to See Own Patients”, whereas the comparison group achieved 51.4% in the measure ”Provider’s Ability to See Own Patients”. It also achieved a higher score of 73.7% (p = 0.12, not significant) in the measure ”Patient’s Ability to See Own Providers” compared to 64.0% in the comparison group. | Average Monthly Probability of Provider’s Ability to See Own Patients and Patient’s Ability to See Own Providers on a range of 0 - 100% (a form of UPC) |
| **Does team-based primary health care improve patients' perception of outcomes? Evidence from the 2007-08 Canadian Survey of Experiences with Primary Health.** | Jesmin S, 2012 | Canada | Retrospective analysis of micro data from the 2007–08 Canadian Survey of Experiences with Primary Health Care | 11521 patient responses in total. 10868 responses were used to evaluate continuity dimension | PCP  Nurse  Nutritionist  Dietician | Aim: ”Regression techniques and propensity score matching method were used to examine the impact of team-based primary care on several process and outcome indicators of primary care” | Primary care was defined as team-based if ”the patient’s regular doctor works in the same office with nurses, dieticians, and nutritionist or if the patient’s regular doctor practices in a Community Health Centre or CLSC”.  Result of Continuity of Care dimension (p < 0.1):   - Team-based PHC: 4379 responses, mean 3.62, S.E. 0.01 - Non-team-based PHC: 6489 responses, mean 3.59, S.E. 0.01   Team-based care was less likely to be associated with the continuity of care in regression results (OR 0.70, p<0.01). Similarly, a negatively significant impact was observed for the continuity of care when calculating the Propensity score (PS). The PS is estimated using a logistic regression of the probability of receiving treatment in team-based PHC on gender, age, household income, household size, education, employment status of the respondents and geographical regions. However both values but became statistically insignificant after considering unobserved selection. | How often taken care by same physician or nurse each time, as rated by patient: Always = 4, Often = 3, Sometimes = 2, Rarely = 1, Never = 0 |
| **Evaluating the implementation of collaborative teams in community family practice using the Primary Care Assessment Tool.** | Moe G, 2019 | 10 WestView Primary Care Network (WPCN) clinics in Alberta, Canada | Cross-sectional, longitudinal study comprising 4 patient telephone surveys between 2007 and 2016 | Adults aged 18 and older with a visit to a family physician in a WPCN clinic at least once in the previous 18 months.  Number of completed surveys:   - 896 in 2007 - 904 in 2010, - 1000 in 2013 - 1800 in 2016 | PCP  Nurse  Pharmacist  Social worker  Mental health professional  Medical office assistants | Aim: ”To examine patients’ perceptions of care outcomes following the introduction  of collaborative teams into community family practices.”  Interventions: ”In 2006, WPCN implemented a decentralized and distributed collaborative  team model, integrating nonphysician health care professionals into member clinics.” | Significant decreases (P < .001) in subscales measuring ongoing care (relational continuity).  Except for in 2010, the 2 clinics scoring highest in the survey were non-participating solo practices; the lowest-scoring clinic was the one with the largest number of physicians. | Primary Care Assessment Tool (PCAT) |
| **General practice staff and patient experiences of a multicomponent intervention for people at high risk of poor health outcomes: a qualitative study.** | Javanparast S, 2024 | Adelaide, South Australia | Qualitative study using semi-structured interviews | 41 practice staff (19 practice managers, 1 Administrative staff, 11 PNs and  10 GPs) and 45 patients | PCP  Practice Manager Practice Nurse  Administrative Staff | Aim: ”The aims of the study were to better understand participant’s (practice staff and patients) perspectives of  the intervention, their views on whether the intervention had improved general practice services, reduced hospital  admissions and finally whether they believed the intervention would be sustainable after the trial had completed.”  Intervention: Multicomponent intervention part of the Flinders QUEST-Trial. The intervention comprised patient enrollment to a preferred General Practitioner (GP) to promote  continuity of care, access to longer GP appointments, and timely general practice follow-up after hospital care  episodes | A team based model of care was viewed as ensuring continuity of care by practice staff. GPs mentioned that continuity could did not only necessarily only with occur with one person but with an entire practice. It was further mentioned that a patient being dependent on only one GP may be a bad thing, since individual GPs are not always present in a practice and if a patient refuses to see another clinician, this may put their health at risk. One GP mentioned that therefore the concept of a preferred GP needs modification. Another GP added that when involving another GP with a patient, they may notice things the patients preferred GP had missed. | Not specified |
| **Patients' experiences with the advanced practice nurse role in Swiss family practices: a qualitative study. Conflict of interest?** | Schönenberger N, 2020 | 4 family practices, Switzerland | Semi-structured interviews | 22 patients who had at least one consultation with an APN | PCP  Advanced practice nurse | Aim: ”Our aim was therefore to explore patients’  experiences with the APN role in Swiss family practices.” | Patients mentioned that APNs may relieve GPs workload thereby ensuring continuity of care. However some patients were reluctant to allow the APN to play the leading role in their care and preferred to receive additional confirmation from their GP. They indicated it was important to them to still be able to speak to the GP even when the APN had done the examination. When the symptoms and complaints were classified as medically severe, patients preferred to consult their GP instead. However, Patients felt that the new role added value to the practice, but also emphasized that the APN and GP were equally important and that the aim was not to replace one profession but for them to complement each other. | Not measured |
| **Modeling Family Medicine Provider Care Team Design to Improve Patient Care Continuity.** | Huang Y, 2023 | Department of Family Medicine at Mayo Clinic in Rochester, Minnesota, United States | Analysis and of 2019 data from the Department of Family Medicine at Mayo Clinic, Rochester, United States | 10 care teams, composed of 36 MDs and 35 nurse practitioners (NPs) / physician assistants (PAs) | PCP  Nurse practitioners  Physician assistant | Aim: ”This study presents a descriptive characterization of  patient care continuity based on provider types and patient management time. […] An optimization model is then used to determine optimal provider mix in a team.” | The study is set in a large primary care practice in which PCPs are grouped into care teams sharing a panel of patients. The optimization model results in the optimal provider assignment, which has an ASOCT percentage (percentage of patient appointments seen by a provider in their own care team) consistently at 62% for all care teams and 3 or 4 physicians (MDs) and NP/PAs in each care team.  The study also noted that having fewer teams increased ASCOT (3% increase when the number of  care teams is reduced by 1). However: ”The purpose of having multiple providers in a care team is to take care of patients for each other while they are away or unavailable […]. One can increase care team continuity by having fewer care teams. However, when more providers are in a care team, there are more patients to study and manage, which could lose the purpose of care team continuity. Hence, the number of provider care teams is based on how many patients a provider is comfortable managing.”  ”Given the discussion above, there might be a curvilinear relationship with the care team size that demonstrates a diminishing return where a care team is no longer efficient in terms of access for care continuity." | ”Care continuity was measured by the percentage of patient appointments seen by a provider in their own care team (ASOCT)” |
| **Implementation of Practice Transformation: Patient Experience According to Practice Leaders.** | Quigley D, 2017 | 14 primary care practices within a large urban Federally Qualified Health Center (FQHC) delivery system in the United States | Semi-structured interviews | 36 interviews with lead physicians (n = 13), site clinic administrators (n = 13), and nurse supervisors (n = 10) | PCP  Nurse Supervisor  Site clinic administrator  Case manager | Aim: ”Examine practice leaders’ perceptions and experiences of how patient-centered medical home (PCMH) transformation improves patient experience." | The study reports that co-locating multiple professions within the same practice meant that patients did not have to be referred to third parties and enabled them to get all care from the same practice. A nurse supervisor noted that team members were able to see and coordinate different aspects of a patients care and were able to provide all of that patients care at once. We interpreted these findings as indicative of improved management continuity.  The study further describes that co-locating team members improved team-based communication. Other factors improving team communication included team planning and coordination, daily huddles, and focusing on customer service. We interpreted these findings as contributing to improved informational continuity. | Not specified |
| **Patients' perceptions of the quality of care after primary care reform: Family medicine groups in Quebec.** | Tourigny A, 2010 | 5 FMGs (family medicine groups) in Quebec, Canada from various settings and types of practice. | Questionnaire-based before-and-after comparison of patients’ perceptions of care at the beginning of family medicine group implementation and 18 months later. | 1046 patients | PCP  Nurse | Aim: ”To evaluate how a primary care reform, which aimed to promote interprofessional and interorganizational collaborative practices, affected patients’ experiences of the core dimensions of primary care.”  Interventions to improve continuity: ”Patients are registered, which improves continuity by linking patients to PCPs and to the professional teams of their FMGs” and ”Electronic patient records within the FMG (although these were not yet available at the time of the study); care pathways for the interdisciplinary followup of patients with chronic diseases, to promote patient empowerment, treatment adherence, and prevention; service agreement with local health agencies to improve patient access to technical and specialized expertise” | The intervention introduced specific measures to improve continuity of care in the FMG team. Overall, concentration of care with the PCP decreased significantly (P < .01), whereas informational and relational continuity significantly increased (P < .001)~~.~~  ”[…] this underlines the difficulty of improving continuity and accessibility simultaneously with a single organizational model and limited resources.” | 1)”Concentration of care”: Patients’ reports on the proportion of visits to the regular physician relative to the total number of visits to any physician of the clinic  2) Modified version of Primary Care Assessment Tool (PCAT) |
| **Scale, scope and impact of skill mix change in primary care in England: a mixed-methods study** | McDermott I, 2022 | 1) Practice manager survey: 1261 general practices in England  2) Case study: 5 general practices in England | 1) Practice manager survey (August–December 2019) exploring factors motivating general practices’ employment decisions  2) In-depth case study of 5 general practices in England (August–December 2019) examining how a broader range of practitioners is experienced by practice staff and patients  3) Analysis of NHS Digital workforce data (2015–19) comparing employment changes and association with outcomes data | 1) Practice manager survey: 1261 practise managers  2) Case study: 38 interviews and 27 observations (totalling 1620 minutes) with practice staff: general practitioners, practice nurses (including ANPs). advanced clinical/care practitioners, physician associates, clinical pharmacists, paramedics, social prescribers, receptionist and practice managers. Focus groups with 29 members of the Patient Participation Groups and 125 patient surveys | PCP  Practice nurse Advanced clinical/care practitioners Physician associates  Clinical Pharmacists Paramedic  Social prescriber Receptionist  Practice managers | Aim: ”Our objective was to conduct a comprehensive study of the scale, scope and impact of changing patterns of practitioner employment in general practice in England. This included an analysis of employment trends, motivations behind employment decisions, staff and patient experiences, and how skill mix changes are associated with outcome measures and costs.” | Patients were generally happy to be seen by newer types of practitioners, but they highlighted the value of continuity and the importance of feeling that practitioners were adequately supported and supervised. Reference was made to the tension between enabling continuity of care and prompt access to appointments with practitioners who might not be their ‘usual’ practitioner. The majority of patients in the survey (65/125, 52%) reported that they received better health care overall when they were always, or mostly, seen by the same practitioner. However in the focus groups, patients felt that skill mix did not always accommodate continuity of care and reported facing difficulties with this and with accessing the same practitioner for follow-up appointments when this was made more difficult by the new access systems. This had a negative impact on patient satisfaction. This deconstruction of relationships with GPs was not without concerns. Focus group patients were concerned about any adverse consequences for patients, particularly older patients, of disrupting long-established relationships. The focus groups did raise concerns about the patient ‘work’ required to understand, access and develop relationships with new practitioners. Given the need for patients to invest time in developing new relationships with newer practitioners, it is useful to note that at sites A and E (both of which were early adopters of skill mix) it was felt that newer practitioners had begun to provide this continuity for some patients, which improved patient satisfaction and increased trust in and acceptance of the newer roles. | Seeing the same practitioner more than once |
| **Creating a safety net for patients with depression in primary care; a qualitative study of care managers' experiences.** | Svenningsson I, 2018 | 23 Primary health care centers in 2 regions of Sweden (12 controls and 11 intervention) | Qualitative, focus group study. | 8 registered nurses | PCP  Nurse | Aim: ”The aim of this study was to explore nurses’ experiences and perceptions of working as care managers at primary care centers.”  Intervention: ”Eight nurses were trained during three days including treatment of depression and how to work as care managers.” | In the study, patients were contacted by care managers (nurses providing support for patients with mild to moderate depression) regularly after the patients first appointment with the GP. The first contact was a face-to-face meeting, followed by 6 telephone calls over 3 months.  The participants perceived that the care manager increased continuity and accessibility of primary health care for patients with depression. The participants perceived that the recurrent telephone calls and the assessment instrument enabled a trusting relationship with the patient. The continuity also helped the care manager to follow the patient’s process as a whole, which prevented a patient from getting lost/falling between contacts.  Participants described their work as a care manager as being a contact person who was available for the patient over time. They saw themselves as a ‘spider in the web’, i.e. a person who had an overview of the patient’s health care contacts, who was an extra link to the GP, and who could coordinate or hasten the care process. Participants also saw themselves as a bridge between the patient and various health care contacts. | Not defined or measured |
| **Rethinking Primary Care Delivery Models: Can Integrated Primary Care Teams Improve Care Experience?** | Duhoux A, 2022 | 6 integrated primary care teams in Quebec, Canada, | Quasi-experimental longitudinal design based on a pre-and-  post administered patient survey at a 2-year interval without a control group | 2186 patients (t0) and 1473 patients (t2) | Medical doctors Nurse practitioners Registered nurses  Licensed practical nurses  Social workers Psychologists Peer helpers | Aim: ”The aim of  this paper is to examine the care experience over time  of patients who have an IPCT [Integrated Primary Care Teams] as their primary source of  care.” | ”The dimension of continuity, which encompassed both relational and management, showed variable scores and very little change over time in all IPCTs. We suggest, therefore, that group practice may be complex and less obviously reflected in care experience.”  The study examined so called Integrated Primary Care Teams (IPCTs) with four defining characteristics: intensive interdisciplinary practice; advanced nursing practice with an expanded role; group practice and increased proximity and availability.  Two questionnaires were used in the study: Q1 was developed for patients for whom IPCT had been the  primary source of care for less than 2 years, focusing on their experience before their first IPCT visit. Q2 was developed for patients for whom the IPCT was the regular source of care for at least 2 years at t0, focusing on their experience in that IPCT.  The study reported a significant increase in the continuity score of Q1 in one site (t0: 7.2, t1: 8.4, p<0.01). As this site had been launched less than 2 years prior to the survey, only Q1 was used at that site. Continuity scores were already high at t0 for most other IPCTs. A slight but significant increase on one site on Q2 was also observed in the continuity dimension (t0: 8.8, t1: 9.0, p<0.01). | 4 items assessing continuity in a questionnaire built by Pineault, adapted from the the Primary Care Assessment  Survey [Safran DG, Kosinski M, Tarlov AR, et al. The primary  care assessment survey: tests of data quality and  measurement performance. Medical Care. 1998; 36(5):  728–739. DOI: <https://doi.org/10.1097/00005650->  199805000-00012  ] and the Primary Care Assessment Tool [Shi L, Starfield B, Xu J. Validating the adult primary care  assessment tool. Journal of Family Practice. 2001; 50(2):  161–175. DOI: <https://doi.org/10.1037/t77102-000>  ].  Continuity of Care was defined as ”The seamless flow in which multiple services are to be provided. These services are continuous if they are  harmoniously linked to each other (management continuity) and when patients are treated continuously by the  same professional or the same team (relational continuity)”, based on the framework provided by Haggerty and Pineault [Pineault R. L’accessibilité et la continuité des services de santé: une étude sur la première ligne au Québec: rapport de recherche soumis aux Instituts de recherche en santé du Canada (IRSC) et à la Fondation canadienne pour la recherche sur les services de santé (FCRSS): Centre de recherche de l’Hôpital Charles Lemoyne [Accessibility and Continuity of Health Services: A Study of the Front Line in Quebec: A Research Report Submitted to the Canadian Institutes of Health Research (CIHR) and the Canadian Health Services Research Foundation (CHSRF): Charles Lemoyne Hospital Research Centre]; 2008. [In French].] [Haggerty JL, Burge F, Lévesque J-F, Gass D, Pineault R, Beaulieu M-D, et al. Operational Definitions of Attributes of Primary Health Care: Consensus Among Canadian Experts. The Annals of Family Medicine. 2007; 5(4): 336–44. DOI: <https://doi.org/10.1370/afm.682>] |
| **Establishing Continuity of Care Through a Team-Based Care Approach: Implementation Challenges.** | Kumar S, 2025 | 2 public primary care clinics in Eastern Singapore | Qualitative study using in-depth semi-structured interviews and focus group discussions | Interviews with 39 HCPs (18 PCPs, 6 nurses, 6 health pals, 9 management team members) and 7 patients | PCPs  Nurses  Health Pals (non-clinical administrative staff) | Aim: ”This study aimed to identify the implementation challenges in delivering CoC through a newly implemented team-based care  model.” | HCPs perceived that team-based care could strengthen relational continuity by helping establishing a stronger relationship with a stable pool of patients, especially for complex chronic patients. This was echoed by patients, who favored seeing the same doctor or team, eliminating the need to constantly repeat their health history to multiple providers.  Management continuity was also perceived as strengthened, as working in a team made the healthcare providers feel more accountable and committed to documenting and managing the patients better as other team members would subsequently see the patient. Task delegation and redistribution was perceived to ensure seamless care coordination.  Barriers to continuity of care were a lack of team stability and unfamiliarity between team members due to frequent rotations caused by manpower shortages. Larger team sizes were further perceived as a barrier to continuity.  Healthcare providers lacked informational continuity due to limited access to electronic health records.  Patients were dissatisfied with receiving conflicting information from different healthcare providers. | Informational, relational and management continuity as described by Haggerty et al.  [Haggerty J, Reid R, Freeman G, Starfield B, Adair C, McKendry R. Conti-  nuity of care: a multidisciplinary review. BMJ. 2003;327:1219–21.] |
